# Interplay between proteostasis pathways and innate immune responses in *Caenorhabditis elegans*

**DOI:** 10.1128/iai.00037-26

**Published:** 2026-04-21

**Authors:** Annesha Ghosh, Jogender Singh

**Affiliations:** 1 Department of Biological Sciences, Indian Institute of Science Education and Research 547342, Mohali, Punjab, India; University of California Merced, Merced, California, USA

**Keywords:** *C. elegans*, endoplasmic reticulum, mitochondria, proteasome, unfolded protein response

## Abstract

Microbial pathogens frequently manipulate host protein homeostasis to undermine immunity by targeting protein synthesis, folding, trafficking, and degradation. Conversely, effective immune responses themselves impose substantial proteostatic demands, as the rapid production of antimicrobial effectors increases the burden on cellular quality-control systems. This bidirectional pressure has likely driven the evolution of surveillance mechanisms that sense disruptions in protein homeostasis as indicators of infection. Using *Caenorhabditis elegans* as a genetically tractable model, recent studies have revealed that perturbations in proteostasis across multiple cellular compartments, including the cytosol, endoplasmic reticulum (ER), mitochondria, proteasome, and extracellular space, are actively integrated with innate immune signaling. Stress-response pathways such as the heat shock response, translational regulation, and the unfolded protein responses of the ER and mitochondria function not only to restore proteome integrity but also to directly shape immune gene expression and pathogen resistance in a context-dependent manner. This review highlights proteostasis as an evolutionarily conserved immune surveillance system, linking cellular stress sensing to host defense and offering broader insights into the coupling of stress adaptation, immunity, and organismal health.

## INTRODUCTION

Proteins are central to nearly every cellular process, yet their functionality depends on a finely tuned balance between synthesis, folding, trafficking, and degradation. Collectively, these processes form the proteostasis network, a multilayered system that preserves protein quality across cellular compartments (1). Disruption of proteostasis leads to the accumulation of misfolded or damaged proteins and imposes proteotoxic stress, a phenomenon extensively studied in the context of aging and neurodegenerative disease. However, cells are not only passive victims of proteostasis imbalance; they possess sophisticated mechanisms to detect and respond to perturbations in protein homeostasis (1, 2).

In parallel, multicellular organisms must constantly defend against microbial pathogens that attempt to hijack host cellular machinery. Pathogens frequently target core proteostasis processes, such as translation, protein folding, secretion, and degradation, to impair host defenses or redirect resources toward pathogen survival (3–5). At the same time, mounting an effective immune response requires rapid and sustained production of immune effectors, placing extraordinary demands on the host’s proteostasis capacity. These opposing pressures raise a fundamental question: how do organisms distinguish between benign proteostatic fluctuations and those indicative of infection?

The nematode *Caenorhabditis elegans* has provided critical insights into this problem. Despite lacking canonical pattern-recognition receptors and specialized immune cells, *C. elegans* exhibits robust innate immune defenses that are tightly coupled to cellular stress responses (6–12). Rather than relying solely on direct pathogen detection, the worm senses disruptions to essential physiological processes, including protein homeostasis, as danger signals (13, 14). Perturbations in proteostasis across the cytosol, endoplasmic reticulum (ER), mitochondria, proteasome, and extracellular environment activate distinct yet interconnected stress-response pathways that converge on immune gene expression.

Importantly, these proteostasis pathways do not function in isolation. Stress responses that evolved to maintain protein quality, such as the heat shock response, unfolded protein responses (UPRs), translational surveillance mechanisms, and extracellular chaperone systems, also shape the magnitude, timing, and outcome of immune activation. In some contexts, proteostasis pathways enhance host defense, while in others, they buffer or restrain immune signaling to limit collateral damage.

This review explores how *C. elegans* integrates proteostasis surveillance with innate immune responses. By examining how different proteostasis pathways intersect with immune signaling under physiological and infectious stress, we aim to highlight general principles governing how organisms leverage protein homeostasis as a central axis of immune regulation.

## PERTURBATIONS IN PROTEIN TRANSLATION AS SURVEILLANCE CUES LINKING PROTEOSTASIS TO INNATE IMMUNITY

Protein translation represents the first committed step in protein synthesis and is therefore central to proteome integrity and cellular homeostasis. The translational machinery operates with a tightly regulated and nonuniform rate of elongation, which critically influences the folding trajectory and fate of nascent polypeptides. Deviations from this optimal rate can be detrimental: excessively rapid translation increases the likelihood of misfolding and aggregation, whereas abnormally slow translation can enhance premature degradation and disrupt proteome balance, ultimately leading to proteotoxic stress (15). As a result, translation occupies a pivotal position at the intersection of proteostasis maintenance and stress signaling.

Consistent with this role, multiple stress response pathways directly modulate translation to buffer proteotoxic stress. The ER UPR (UPR^ER^) and the integrated stress response pathways attenuate global protein synthesis, thereby reducing the influx of newly synthesized proteins into already burdened folding environments (16–18). At the same time, inhibition of protein translation itself has emerged as a conserved signal of pathogen attack, capable of triggering innate immune responses across diverse organisms, including mammals, insects, and nematodes (19–25). Translation inhibition thus occupies a dual role: it functions both as a protective proteostatic adjustment and as an immune surveillance cue.

This duality is particularly evident during host-pathogen interactions. Because effective immune responses require the rapid synthesis of antimicrobial and stress response proteins, host translation is a prime target for microbial virulence strategies (22). In response, hosts have evolved mechanisms to detect disruptions in translation as indicators of infection. In *C. elegans*, the bacterial pathogen *Pseudomonas aeruginosa* employs multiple strategies to inhibit host translation, including elongation blockade by exotoxin A and cleavage of the ribosomal decoding center (19, 20, 26). These perturbations are not merely tolerated but actively sensed by the host to initiate immune defense programs.

A well-characterized response to translational inhibition in *C. elegans* involves the bZIP transcription factor ZIP-2. Under basal conditions, ZIP-2 translation is suppressed by an upstream open reading frame (uORF) overlapping the main coding sequence (19). Upon elongation inhibition, a proposed +1 frameshift event is thought to fuse the uORF and main ORF, enabling production of functional ZIP-2 protein (27). Although indirect evidence supports this model, the predicted fused ZIP-2 product has not yet been directly detected (27), and further biochemical validation will be required. During *P. aeruginosa* infection, ZIP-2 heterodimerizes with the CCAAT enhancer-binding protein CEBP-2 to induce immune effectors such as *irg-1*, *irg-2*, and *pgp-5* (24, 28). In parallel, a ZIP-2-independent pathway has been identified in which the Tribbles kinase ortholog NIPI-3 functions as a sensor of translational stress to activate innate immunity, a response that is negatively regulated by the transcription factor CEBP-1 (29). Together, these pathways highlight how translation perturbations are integrated into immune signaling networks.

**Fig 1:**
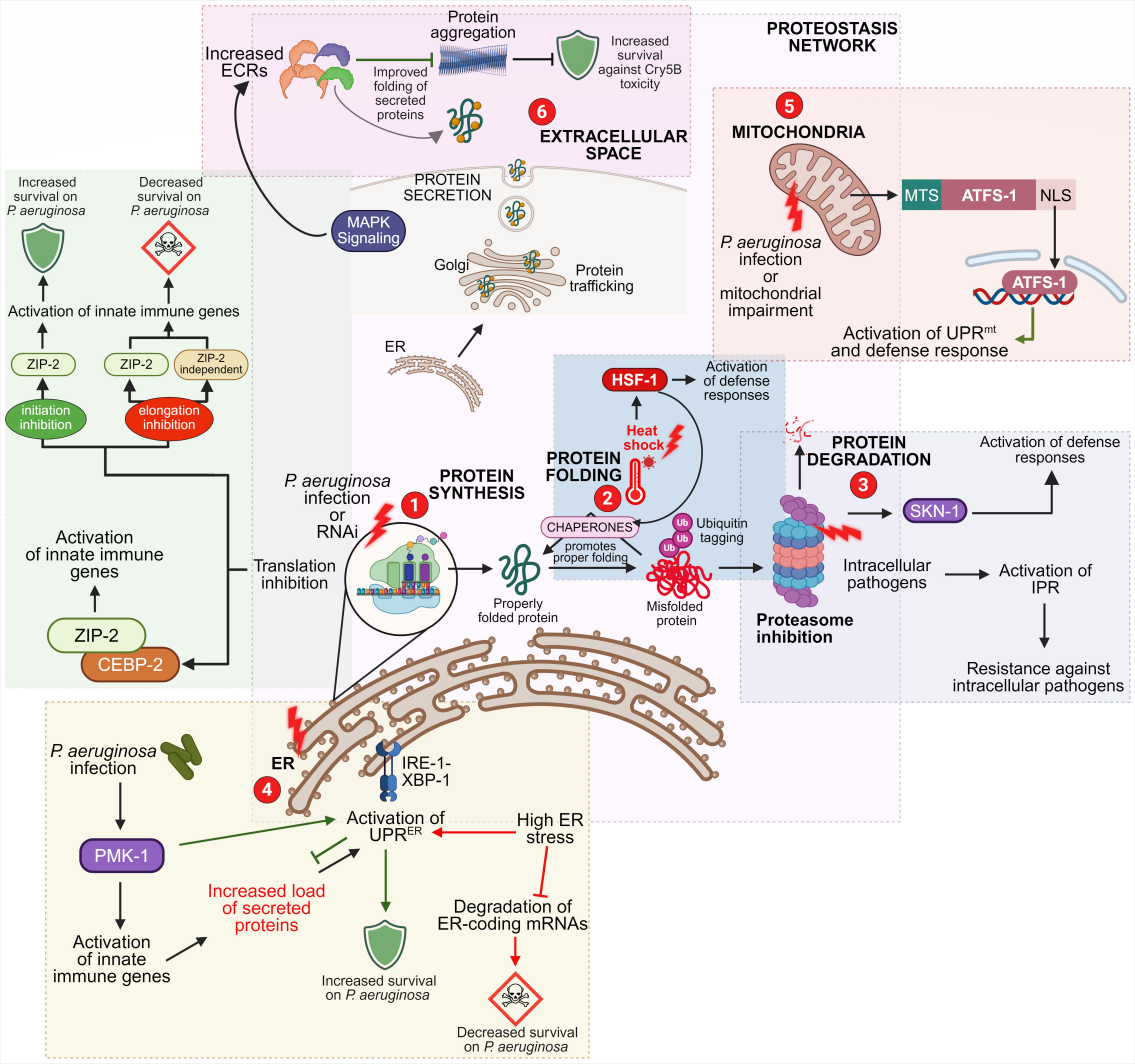
Interplay between proteostasis pathways and innate immune responses in *C. elegans*. (1) Inhibition of protein translation activates ZIP-2-mediated innate immune defenses. (2) Heat shock induces HSF-1-dependent expression of heat shock proteins, conferring protection against pathogenic infection. (3) Inhibition of proteasomal activity activates SKN-1-mediated defense responses and simultaneously induces the intracellular pathogen response (IPR), which enhances resistance to intracellular pathogens. (4) Elevated endoplasmic reticulum (ER) stress, resulting from increased protein misfolding or heightened secretory load during immune activation, triggers the UPR^ER^, which promotes survival during *P. aeruginosa* infection. However, excessive ER stress also suppresses mRNAs encoding ER-targeted proteins, including several immune effectors, thereby reducing host survival on *P. aeruginosa*. (5) *P. aeruginosa* infection or mitochondrial dysfunction activates the UPR^mt^ and associated defense programs through the transcription factor ATFS-1. (6) Extracellular proteostasis is regulated by extracellular regulators (ECRs) under the control of KGB-1 and JNK-1 MAPK signaling pathways. ECRs limit extracellular protein aggregation and protect against Cry5B toxin-induced damage.

An important unresolved question has been whether immune gene induction under translation inhibition translates into functional host protection. Although transcriptional upregulation of immune effectors is robust in response to impaired translation, it has remained unclear whether sufficient immune effectors can be synthesized when overall translational capacity is compromised. Addressing this, a recent systematic analysis examined host survival during *P. aeruginosa* infection when distinct translation stages were selectively inhibited. Although both initiation and elongation inhibition activated ZIP-2-dependent immune responses, the physiological outcomes diverged markedly (Fig. 1). Inhibition of translation initiation enhanced host survival, whereas elongation inhibition, despite triggering additional ZIP-2-independent immune pathways, proved detrimental, likely due to excessive stress and immune overload (21).

Intriguingly, earlier work had reported a different pattern of ZIP-2 dependence: immune gene induction following elongation inhibition was described as ZIP-2-dependent, whereas induction triggered by initiation inhibition appeared ZIP-2-independent (19). This apparent discrepancy may arise from differences in experimental design. The latter study based its conclusions primarily on the behavior of a single immune reporter gene, *irg-1* (19), whereas the former relied on transcriptome-wide analysis of *zip-2* mutants subjected to translation initiation inhibition (21). Notably, *irg-1* expression under initiation inhibition appears partially or fully independent of ZIP-2 (21), suggesting that single-gene analyses may not fully capture the broader regulatory architecture. Accordingly, global transcriptomic profiling likely provides a more comprehensive and mechanistically informative view of how stage-specific translational perturbations shape immune gene networks and functional outcomes.

Complementary insights come from studies examining how stage-specific translation inhibition differentially impacts proteostasis. Inhibiting translation initiation reduces the burden of newly synthesized proteins and protects against heat stress and age-associated protein aggregation, whereas elongation inhibition confers resistance to proteasome dysfunction but fails to alleviate age-related aggregation phenotypes (30). These findings underscore that the proteostasis network responds distinctly depending on which stage of translation is disrupted, with important consequences for immune activation and organismal fitness.

Collectively, these studies establish protein translation as a critical surveillance node at the interface of proteostasis and innate immunity. Perturbations in translation can act both as a cause and a consequence of cellular stress, and the immune outcomes depend sensitively on the nature and stage of translational disruption. The mechanistic basis underlying these differences, particularly how distinct proteostasis pathways shape immune signaling when translation initiation versus elongation is inhibited, remains an important open question. Addressing this complexity will be essential for understanding how cells discriminate between adaptive and pathological stress responses during infection.

## CYTOSOLIC CHAPERONES COORDINATE PROTEIN FOLDING AND HOST IMMUNITY

Following protein synthesis, the correct folding of nascent polypeptides is primarily orchestrated by the cytosolic heat shock protein (HSP) chaperone network. The existence of this system was first revealed through classical studies in *Drosophila*, where exposure to elevated temperatures induced characteristic chromosomal puffing patterns that corresponded to the rapid transcriptional activation of heat shock genes (31–33). Subsequent work established HSPs as molecular chaperones essential for protein folding, complex assembly, and the prevention of aggregation under both basal and stress conditions (34, 35). Because protein misfolding represents a fundamental threat to cellular viability, the heat shock response constitutes a central pillar of cytosolic proteostasis.

In *C. elegans*, several bacterial pathogens induce protein aggregation in the intestine (36, 37). Heat shock factor 1 (HSF-1)-dependent induction of HSPs, including HSP-12.6 and HSP-16.41, limits pathogen-induced protein aggregation (36). These protective effects also require the FOXO transcription factor DAF-16. Beyond maintaining proteome integrity, the heat shock response plays a critical role in regulating immune function. A brief exposure of adult worms to heat stress confers enhanced resistance to subsequent infection by pathogenic bacteria, an effect mediated by HSF-1-dependent HSP induction (11, 36, 38). Several HSPs, including HSP-16.20, HSP-16.21, HSP-16.1, and HSP-90, are required for improved survival following pathogen exposure after heat shock (11). Mechanistically, HSF-1 promotes host defense by regulating the nuclear localization of DAF-16, thereby integrating stress adaptation with immune gene regulation (39). Although DAF-16 is known to control HSPs expression (40), it remains unclear whether its protective role during infection in this context is mediated primarily through HSP induction or through the activation of distinct immune effectors. Importantly, HSF-1 is also required for effective immunity under diverse infection conditions independent of acute thermal stress, indicating that its contribution to host defense extends beyond the canonical heat shock response (41, 42).

Strikingly, the immunological consequences of heat shock can be long-lasting. Early-life exposure to elevated temperatures enhances immune competence in adult animals through sustained activation of the p38 MAPK pathway, with these protective effects maintained by changes in histone acetylation (43). Subsequent work demonstrated that this immune memory requires activation of the UPR^ER^, which shapes immune output by inhibiting ribosome biogenesis and revealing functional crosstalk between the ER and the nucleolus (44). These findings highlight how transient proteostatic challenges can imprint durable immune states through epigenetic and inter-organelle signaling mechanisms.

Recent studies have further complicated the relationship between cytosolic proteostasis and immunity. Unexpectedly, *hsf-1* loss-of-function mutants exhibit elevated expression of innate immune genes at the day one adult stage. This immune activation arises from compensatory engagement of alternative stress response pathways, including the UPR^ER^, the SKN-1/NRF1/2 oxidative stress pathway, and GATA factor-dependent immune programs mediated by ELT-2 (45). These observations suggest that disruption of the heat shock response does not simply weaken host defense but instead reshapes immune signaling through stress response rewiring, underscoring the plasticity of proteostasis-immunity networks.

Cytosolic proteostasis is also intimately linked to host defense against intracellular pathogens. Infection by several intracellular microbes triggers an intracellular pathogen response (IPR) characterized by induction of genes involved in protein homeostasis and ubiquitin-mediated degradation (46, 47). Genetic activation of the IPR confers enhanced thermotolerance, reinforcing the bidirectional relationship between proteostasis and stress resilience (48). Notably, the IPR-associated cullin protein CUL-6, a component of a Skp-Cullin-F-box (SCF) ubiquitin ligase complex, promotes thermotolerance by targeting the molecular chaperone HSP-90 for degradation (49). This finding reveals a regulatory mechanism in which selective remodeling of the chaperone network enhances organismal adaptation to proteotoxic stress.

Consistent with these findings, infection with the Orsay virus improves thermotolerance in *C. elegans*, suggesting that activation of immune and proteostasis pathways during intracellular infection can confer cross-protection against thermal stress (50). Conversely, prior exposure to heat shock reduces Orsay virus infection, indicating that elevated proteostatic capacity can enhance antiviral defense (51). Collectively, studies of both intracellular and extracellular pathogens support a reciprocal relationship between thermotolerance and immunity, in which cytosolic chaperone systems function not only as guardians of protein folding but also as dynamic regulators of host defense programs.

## PROTEOTOXIC INSULTS ARISING FROM DISRUPTION OF PROTEASOMAL ACTIVITY ELICIT DISTINCT IMMUNE RESPONSES

A substantial fraction of cellular proteins is ultimately removed by the ubiquitin-proteasome system (UPS), which ensures proteome integrity through selective recognition and degradation of damaged, misfolded, or surplus proteins. Substrates destined for degradation are tagged with ubiquitin chains, enabling their engagement by the proteasome and subsequent proteolysis (52). Under conditions of proteotoxic stress, such as heat shock, oxidative damage, or the accumulation of misfolded and aggregated proteins, cells increase proteasome abundance and activity to accelerate protein clearance and restore homeostasis (53). Beyond its role in protein quality control, the UPS also functions as a critical component of host defense by targeting intracellular pathogens for ubiquitin-mediated recognition and facilitating their autophagic clearance (54, 55).

Given its central role in cellular surveillance, it is not surprising that pathogens have evolved multiple strategies to evade, hijack, or suppress the host UPS (56–58). Conversely, hosts have evolved mechanisms to sense disruptions in proteasomal function as indicators of infection or cellular damage, triggering compensatory stress responses and immune activation. In this context, impaired UPS activity is not merely a proteostatic defect but also a signal that can trigger immune reprogramming.

In *C. elegans*, the integration of UPS function with immune signaling is evident in systemic stress responses triggered by genotoxic and proteotoxic insults. DNA damage in the germline induces organism-wide stress resistance through activation of innate immune pathways, with enhanced proteasomal activity acting as a downstream effector to restore proteostasis and promote survival (59). Conversely, direct inhibition of the proteasome activates a compensatory transcriptional response mediated by the NRF1/2 homolog SKN-1, which induces proteasome subunit genes in a “bounce-back” mechanism aimed at restoring degradative capacity (60, 61). Notably, SKN-1 also promotes immune defense programs against extracellular pathogens, linking proteasomal surveillance to innate immunity (62, 63). Distinct SKN-1 isoforms appear to specialize in different aspects of this crosstalk: SKN-1C protects against extracellular pathogen infection, whereas SKN-1A suppresses immunity to intracellular pathogens and oomycetes ([64, 65] and see below). Indeed, *skn-1a* loss-of-function mutants display increased resistance to microsporidia and oomycetes (64).

Proteasome dysfunction further engages additional immune-regulatory transcription factors. Inhibition of proteasomal activity activates the intestinal GATA transcription factor ELT-2, a central regulator of immune gene expression in *C. elegans* (66, 67). Similarly, disruption of the UFD-1-NPL-4 complex, which targets ER-associated misfolded proteins for proteasomal degradation, triggers ELT-2-dependent immune responses (68). Intriguingly, while these immune responses triggered by UFD-1-NPL-4 complex inhibition can be detrimental in otherwise healthy animals, likely due to chronic stress and immune overactivation, they improve the survival of immunocompromised worms during pathogen exposure. These studies show that perturbation of different proteostasis pathways linked with the proteasome might converge on ELT-2 signaling. Nonetheless, these findings highlight a context-dependent trade-off between proteostasis restoration and immune activation.

Beyond its canonical degradative functions, the proteasome can also influence immunity through non-proteolytic mechanisms. The proteasomal subunit RPT-6 has been shown to physically interact with ELT-2 in the nucleus, thereby promoting ELT-2-dependent immune gene expression independently of proteolysis (69). This unexpected role underscores the versatility of proteasomal components as signaling platforms and reinforces the bidirectional nature of UPS-immune crosstalk.

Pathogens, in turn, exploit this regulatory axis to suppress host defense. The intracellular bacterium *Burkholderia pseudomallei* uses a type III secretion system to promote degradation of ELT-2 via the host UPS, thereby attenuating immune responses in *C. elegans* (70). This strategy exemplifies how microbial manipulation of proteasomal pathways can directly undermine host immune transcriptional programs.

The importance of the UPS in immune defense is further evident during infection with natural pathogens of *C. elegans*, including oomycetes and intracellular microbes such as *Nematocida parisii* and Orsay virus (47, 71, 72). These infections elicit tissue-specific yet partially overlapping transcriptional programs, some of which overlap with those induced by proteasome inhibition (47, 64, 71–73). However, proteasome inhibition uniquely triggers SKN-1A-dependent expression of proteasome subunit genes, a response not observed during *N. parisii* or oomycete infection (64, 73). SKN-1A promotes resistance to proteotoxic stress (61), and its inhibition induces proteotoxic stress that activates the IPR and oomycete response genes, thereby enhancing resistance to these infections (64). Although infection with intracellular pathogens or oomycetes does not induce proteasome subunit genes, it stimulates expression of components of an SCF ubiquitin ligase complex, including multiple *skr* genes and *cul-6* (64, 73). While this SCF complex plays only a modest role in protecting from intracellular pathogens, it enhances proteostasis (46–48), thereby establishing a reciprocal link between intracellular infection and protein quality control.

Together, these studies establish the UPS as a central hub linking proteostasis and innate immunity in *C. elegans*. Proteasomal dysfunction is actively monitored by the host and translated into immune responses through multiple transcriptional regulators, while immune signaling reciprocally shapes proteasomal activity to enhance stress resilience. This bidirectional crosstalk enables *C. elegans* to integrate intracellular damage signals with pathogen defense, ensuring adaptive responses to both proteotoxic stress and infection.

## ACTIVATION OF UPR^ER^ ACTS AS BOTH A DRIVER AND A CONSEQUENCE OF INNATE IMMUNITY

The ER plays a central role in proteostasis by coordinating protein synthesis, folding, and trafficking within the secretory pathway. ER-resident chaperones and folding enzymes ensure that nascent proteins achieve their correct conformations before being transported to their functional destinations. Under homeostatic conditions, misfolded or unassembled proteins are either refolded with the assistance of chaperones or targeted for degradation through ER-associated degradation, thereby preventing the accumulation of nonfunctional or toxic species (74). However, diverse physiological and pathological stresses can overwhelm ER folding capacity, leading to the accumulation of unfolded or misfolded proteins and the induction of ER stress.

To restore ER homeostasis, eukaryotic cells activate the UPR^ER^, a conserved signaling network comprising three principal branches: the PERK, IRE-1, and ATF-6 pathways. These sensors are normally maintained in an inactive state through association with the ER chaperone BiP/GRP78 but become activated upon BiP dissociation during ER stress. Collectively, UPR^ER^ signaling reduces global protein synthesis to limit further ER burden while inducing chaperones, folding enzymes, and quality-control factors to enhance ER folding capacity and degradative output (17).

Innate immune activation during pathogen infection imposes a substantial burden on the ER and can itself provoke ER stress (75–77). Effective pathogen clearance requires robust production of immune effector molecules, many of which are secreted proteins, including antimicrobial peptides and lysozymes (76). Consequently, innate immune activation imposes a significant load on the ER folding and trafficking machinery (78). In mammalian systems, UPR^ER^ signaling is well established as a key regulator of immune cell function, particularly in professional secretory cells (78–81). Analogously, pathogen infection in *C. elegans* induces ER stress in intestinal cells, necessitating adaptive UPR^ER^ activation for host survival (77, 82).

Consistent with this idea, the IRE-1-XBP-1 branch of the UPR^ER^ is essential for protection against pathogen-induced stress in *C. elegans* (82). Importantly, activation of XBP-1 during infection requires signaling through the PMK-1/p38 MAPK pathway, a central regulator of innate immunity. XBP-1 becomes essential only under conditions where PMK-1-dependent immune responses are engaged, revealing a functional coupling between immune activation and ER stress adaptation (82). Further studies demonstrated that PMK-1-driven immune gene expression substantially contributes to ER stress by increasing the secretory load of immune effectors (82, 83). In this context, XBP-1 functions as a protective factor that buffers the physiological cost of immune activation, ensuring that enhanced secretion does not compromise ER integrity (Fig. 1).

In addition to mitigating stress caused by immune-driven secretion, the UPR^ER^ can directly shape immune gene expression. Recent work has shown that UPR^ER^ signaling regulates innate immune responses in a temperature-dependent manner, further highlighting its role as an active modulator of host defense rather than a passive stress response (44). Consistent with this, genetic or pharmacological activation of the UPR^ER^ enhances host survival during pathogen infection, reinforcing the protective role of ER stress adaptation in immunity (77, 84–86).

ER proteostasis is also regulated through mechanisms beyond canonical UPR signaling (87). Under conditions of severe ER stress, cells can selectively degrade mRNAs encoding ER-targeted proteins, thereby reducing the influx of nascent polypeptides into the ER and alleviating folding stress (88). In *C. elegans*, this mechanism leads to the downregulation of transcripts encoding secreted proteins, including immune effectors, effectively dampening immune output under extreme stress conditions (89). This response underscores a critical trade-off between sustaining immune defense and preserving ER integrity.

Collectively, these studies reveal that UPR^ER^ activation can function both upstream and downstream of innate immune signaling. ER stress can act as a trigger for immune responses, while immune activation itself generates ER stress that necessitates UPR^ER^ engagement. This bidirectional relationship positions the ER as an important node in proteostasis-based immune surveillance, enabling organisms to dynamically balance secretory demand with ER homeostasis maintenance during infection.

## ACTIVATION OF THE UPR^MT^ ELICITS BROAD PROTECTIVE DEFENSE MECHANISMS

Mitochondria play a central role in cellular metabolism and proteostasis and activate a dedicated unfolded protein response (UPR^mt^) upon sensing mitochondrial dysfunction. Mitochondrial stress can arise from diverse sources, including mutations in the mitochondrial genome, excessive production of reactive oxygen species during oxidative phosphorylation, accumulation of misfolded or unassembled mitochondrial proteins, and exposure to bacterial toxins (23, 90–92). Such perturbations compromise mitochondrial function, threaten organismal fitness, and contribute to aging. To counteract these insults, cells engage the UPR^mt^, a transcriptional program that induces mitochondrial chaperones, proteases, and quality-control factors to restore proteostasis and maintain mitochondrial function (93).

Because the vast majority of mitochondrial proteins are encoded by nuclear genes, synthesized in the cytosol, and imported into mitochondria, effective communication between mitochondria and the nucleus is essential for mitochondrial homeostasis (94). In *C. elegans*, UPR^mt^ activation is primarily coordinated by two transcription factors: the bZIP protein ATFS-1 and the homeobox transcription factor DVE-1. ATFS-1 contains both a mitochondrial targeting sequence and a nuclear localization sequence. Under homeostatic conditions, ATFS-1 is efficiently imported into mitochondria and degraded. However, when mitochondrial import efficiency is compromised under stress, ATFS-1 accumulates in the nucleus, where it activates transcription of UPR^mt^ genes (95). In parallel, DVE-1 redistributes to chromatin and associates with promoters of mitochondrial chaperone genes, reinforcing the transcriptional UPR^mt^ program (96).

In addition to sensing intrinsic mitochondrial dysfunction, the UPR^mt^ functions as a surveillance mechanism during bacterial infection. Pathogenic microbes can disrupt mitochondrial homeostasis either directly, through toxins and metabolic interference, or indirectly, through host immune activation. Supporting this notion, infection of *C. elegans* with *P. aeruginosa* induces nuclear accumulation of ATFS-1, indicating activation of the UPR^mt^. This response is functionally important, as loss of *atfs-1* renders animals highly susceptible to *P. aeruginosa* infection, demonstrating that UPR^mt^ signaling contributes directly to host defense (12).

Given its protective role, it is perhaps unsurprising that pathogens have evolved strategies to suppress UPR^mt^-mediated immunity. *P. aeruginosa* exploits the host bZIP transcription factor ZIP-3, a negative regulator of the UPR^mt^, to dampen mitochondrial stress responses and undermine immune defense (97). In addition, the *P. aeruginosa* enzyme FadE2, an acyl-CoA dehydrogenase, disrupts host mitochondrial metabolism by specifically perturbing valine and leucine catabolism, thereby suppressing UPR^mt^ activation and altering host energy homeostasis (98). These findings illustrate that pathogens target not only mitochondrial function itself but also the regulatory architecture of the UPR^mt^ to evade immune surveillance.

Importantly, mild mitochondrial perturbations are sufficient to activate ATFS-1-dependent UPR^mt^ signaling, which leads to upregulation of innate immune genes and confers resistance to *P. aeruginosa* infection independently of the canonical PMK-1/p38 MAPK pathway (99). Indeed, multiple studies have demonstrated that genetic activation of the UPR^mt^ enhances resistance to bacterial pathogens (100, 101). Moreover, pharmacological induction of the UPR^mt^ has been shown to protect against bacterial infection, suggesting that this pathway may represent a tractable therapeutic target for enhancing host defense (102, 103). Together, these findings position the UPR^mt^ as a central mitochondrial surveillance mechanism that integrates proteostasis, metabolism, and innate immunity.

## EXTRACELLULAR PROTEOSTASIS AS AN EMERGING REGULATOR OF INNATE IMMUNITY

A large fraction of the proteome is secreted to function extracellularly as signaling molecules, immune effectors, transport proteins, and enzymes. Once outside the cell, these proteins are no longer protected by intracellular quality control systems and instead rely on a limited set of extracellular regulators (ECRs) to maintain proteome stability (104, 105). Disruption of extracellular proteostasis has severe pathological consequences and is linked to numerous human diseases characterized by extracellular protein aggregation (106, 107), including Alzheimer’s disease, where secreted amyloidogenic proteins form toxic oligomers and fibrils (108).

The ER exerts upstream control over extracellular proteostasis by regulating the folding, quality control, and secretion of proteins destined for export. Accordingly, ER stress responses influence the composition and stability of the extracellular proteome (109). Activation of the UPR^ER^ limits the secretion of aggregation-prone proteins and promotes the extracellular release of chaperones such as ERdj3, which can suppress protein aggregation outside the cell (110). These findings underscore a functional link between ER proteostasis and extracellular protein quality control.

Recently, *C. elegans* has been leveraged as an *in vivo* model to monitor extracellular protein aggregation. A genetic screen identified regulators whose knockdown accelerates extracellular aggregation, leading to the discovery of 57 extracellular regulator (ECR) genes essential for maintaining extracellular proteome stability (111, 112). Notably, ECRs were enriched for genes induced during infection and were regulated by conserved stress-activated MAPK pathways, including KGB-1 and JNK-1, both implicated in immune defense. Functionally, overexpression of ECRs enhanced resistance to Cry5B, a pore-forming toxin produced by *Bacillus thuringiensis*, demonstrating that strengthening extracellular proteostasis directly improves host survival during pathogenic challenge (Fig. 1).

Together, these findings establish extracellular proteostasis as an active and inducible component of innate immunity rather than a passive extension of intracellular quality control. By stabilizing secreted proteins and mitigating extracellular proteotoxic stress, extracellular proteostasis regulators contribute to systemic host defense. However, key questions remain regarding how extracellular proteostatic disturbances are sensed, how these signals intersect with canonical immune pathways, and how extracellular and intracellular stress responses are coordinated during infection (112).

## CONCLUSIONS AND FUTURE PERSPECTIVES

Proteostasis is fundamental to organismal health, longevity, and disease resistance. Age-associated decline in protein quality control and stress adaptation has long been viewed primarily through the lens of neurodegeneration and chronic proteotoxic disorders. However, work in *C. elegans* has reframed this paradigm by revealing that proteostasis disruption is not solely pathological. Instead, perturbations in protein folding, trafficking, translation, and degradation can be actively sensed as danger signals that engage innate immune defenses. This review highlights how proteostasis surveillance across multiple cellular compartments functions as an integral component of host defense, enabling organisms to detect and respond to pathogenic threats that directly or indirectly disrupt protein homeostasis.

Proteins are continuously monitored throughout their lifecycle by compartment-specific quality control systems in the cytosol, ER, mitochondria, proteasome, and extracellular space. Pathogens frequently target these systems to subvert immunity, while hosts have evolved stress-response pathways that both restore proteome balance and initiate immune signaling. Studies in *C. elegans* demonstrate that unfolded protein responses, proteasomal surveillance, translational control, and extracellular proteostasis are tightly coupled to conserved immune pathways and longevity programs, collectively shaping resistance to a broad range of intra- and extracellular pathogens.

A recurring theme emerging from these studies is context dependence. While mitochondrial stress and UPR^mt^ activation generally confer robust protection during infection, UPR^ER^ signaling can exert divergent effects depending on physiological state, genetic background, and environmental conditions. Similarly, perturbations at distinct stages of protein translation elicit qualitatively different proteostatic and immune outcomes, raising fundamental questions about how translational control interfaces with immune surveillance. Dissecting whether these responses converge on shared signaling nodes or represent parallel defense strategies remains an important challenge.

Extracellular proteostasis represents a particularly underexplored frontier. The discovery that extracellular quality-control mechanisms actively contribute to systemic immunity opens new avenues to study how secreted protein homeostasis is coordinated with intracellular stress responses. Future work will be essential to define how proteostatic signals are integrated across compartments and how this integration shapes immune specificity, robustness, and long-term organismal fitness.

Together, insights from *C. elegans* position proteostasis not merely as a maintenance system but as an adaptive surveillance network. Understanding how organisms exploit proteotoxic stress to trigger protective immunity may reveal conserved principles relevant to infection, aging, and proteostasis-related diseases in higher organisms.
